# Trends in colorectal cancer screening compliance and incidence among 60‐ to 74‐year‐olds in China

**DOI:** 10.1002/cam4.7133

**Published:** 2024-04-17

**Authors:** Mingqing Zhang, Yongdan Zhang, Lu Guo, Lizhong Zhao, Haoren Jing, Xiao Yang, Wen Zhang, Yong Zhang, Zhenguo Nie, Siwei Zhu, Shiwu Zhang, Xipeng Zhang

**Affiliations:** ^1^ Department of Colorectal Surgery Tianjin Union Medical Center Tianjin China; ^2^ Nankai University School of Medicine Nankai University Tianjin China; ^3^ Tianjin Institute of Coloproctology Tianjin China; ^4^ The Institute of Translational Medicine, Tianjin Union Medical Center of Nankai University Tianjin China; ^5^ Center for Applied Mathematics, Tianjin University Tianjin China; ^6^ Endoscopy Center, Tianjin Union Medical Center Tianjin China; ^7^ Department of Pathology Tianjin Union Medical Center Tianjin China

**Keywords:** colonoscopy, compliance, detection rate

## Abstract

**Background:**

Compliance with colonoscopy among elderly individuals participating in colorectal cancer (CRC) screening programs is unsatisfactory, despite a high detection rate of bowel‐related diseases. In this study, our aim was to analyze the impact of risk factors on the trends of compliance and detection rates in colonoscopy among high‐risk individuals aged 60–74.

**Methods:**

A retrospective study was conducted on the high‐risk individuals aged 60–74 participating in the 2021 CRC screening program in Tianjin, China. Logistic regression analyses, including both univariate and multivariate analyses, were performed to explore the impact of different risk factors on colonoscopy compliance among the high‐risk individuals. Besides, the study investigated the influence of various risk factors on the detection rates of bowel‐related diseases among the high‐risk individuals who underwent colonoscopy.

**Results:**

A total of 24,064 high‐risk individuals were included, and 5478 individuals received a free colonoscopy, with an overall compliance of 22.76%. Among them, the adenoma detection rate was 55.46%. Males and individuals with a positive FIT had high compliance and detection rates for CRC, advanced adenomas (AA), advanced colorectal neoplasia (ACN), and colorectal neoplasm (CN). Individuals aged 70–74 were associated with low compliance but high CRC, ACN, and CN detection rates. Individuals who reported a history of chronic constipation, bloody mucous, and CRC in first‐degree relative showed high compliance but no significantwere associated with the detection rates of CRC, AA, and CN.

**Conclusion:**

This study reported several risk factors associated with the screening behaviors for CRC. Patterns and trends in CRC, AA, ACN, and CN compliance and detection rates correlate with risk factors.

## 1 INTRODUCTION

The incidence and mortality of colorectal cancer (CRC) have witnessed a significant increase, and this trend is also evident in China.^1^, ^2^ Specifically, in China, incidence (average annual percentage change (AAPC) 1.6, 95% CI 1.3–1.9) and mortality (AAPC 1.3, 95% CI 0.9–1.7) are increasing rapidly in males; the trend in female mortality is slightly increasing (AAPC 0.6, 95% CI 0.0–1.2) and incidence is stable.^2^ CRC screening could be used as an effective tool to reduce the incidence rate and mortality of CRC.^3^, ^4^ Regrettably, there are still significant differences in CRC screening strategies and performances.

Colonoscopy plays a significant role in CRC screening.^5^ However, the compliance varies greatly, range from about 20% to over 60%.^6^, ^7^, ^8^, ^9^, ^10^, ^11^, ^12^ Inadequate compliance with colonoscopy is affected by many factors, such as the invasiveness of colonoscopy, the requirements of intestinal preparation, and insufficient awareness of CRC screening. In addition, regional and ethnic differences may also be the influencing factors.^13^, ^14^ Therefore, it is critical to try to improve compliance by implementing a variety of programs.^15^ Additionally, studies have found that the combined use of FIT and risk stratification scores could facilitate the sensitivity of detecting advanced tumors without significantly increasing the workload of colonoscopy.^16^, ^17^, ^18^ The screening program in china that combines high‐risk factor questionnaire (HRFQ) with fecal immunochemical test (FIT)^19^ and subsequent colonoscopy is also a combined strategy. Despite the widespread application of this CRC screening program, there is still not enough evidence regarding its effectiveness and convenience. Therefore, there is a need to analyze the compliance and detection rate trends of the relevant risk factors in this program.

It has been previously found that colonoscopy compliance declines^20^ with age, while the incidence rate of bowel‐related diseases such as CRC increases with age. Consequently, it is critical in the screening process to weigh and recommend whether the elderly high‐risk populations should undergo colonoscopy. A retrospective study of CRC screening data in Tianjin, China, including participants aged 60–74 years in 2021, was conducted in the current study. The differences in colonoscopy compliance among different high‐risk groups were analyzed. Additionally, the value of various high‐risk factors in detecting bowel‐related diseases among high‐risk populations undergoing colonoscopy was investigated. This work aims at providing some basis for optimizing CRC screening strategies.

## 2 METHODS

### 2.1 Study design and populations

A screening program combining HRFQ and FIT tests was launched in Tianjin, China in 2012.^21^ The screening program is open to all individuals within the specified age group. Individuals will undergo both HRFQ and FIT tests, and if either test yields a positive result, they will be identified as high‐risk individuals and offered a free colonoscopy examination.

The recommended positive definition of HRFQ is considered to meet any of the following criteria: (1) a history of CRC in a first‐degree relative; (2) a history of cancer or intestinal polyps; (3) individuals with two or more of the following: a history of chronic constipation, a history of chronic diarrhea, a history of bloody mucous stools, adverse life events (e.g., divorce and death of a close relative), a history of chronic appendicitis or appendectomy, and a history of chronic cholecystitis or gallstones. And a positive HRFQ result indicates an increased risk of CRC. The HRFQ has been used in CRC screening widely in China.^22^, ^23^, ^24^, ^25^, ^26^, ^27^

**FIGURE 1:**
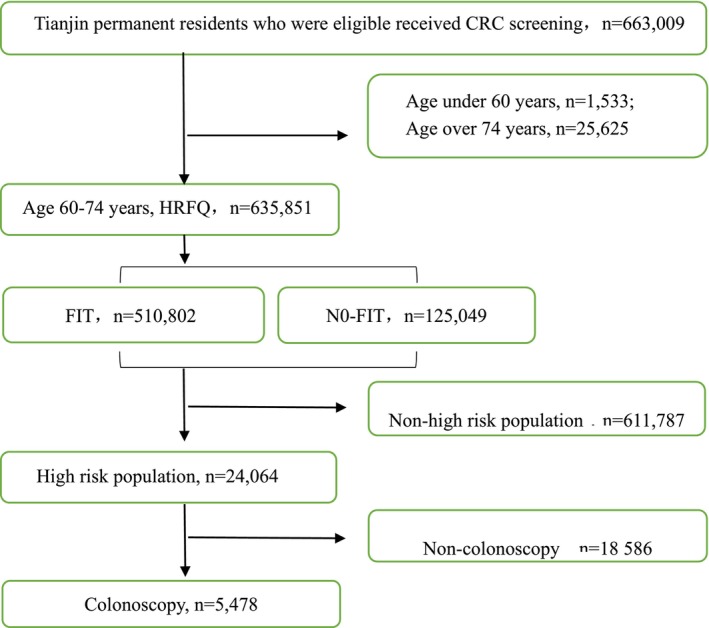
Study population screening flow chart.

In 2021, the primary populations screened were those aged 60–74. Our study included a total of 24,064 high‐risk individuals, with exclusion criteria applied to those outside the specified age group, those with missing data, and those who had previously undergone primary screening. Figure 1 illustrates the flow chart depicting the inclusion and arrangement of the individuals.

### 2.2 Risk factors and demographic characteristics

All variables in the HRFQ and FIT were considered risk factors for this study. In addition, the database also recorded demographic characteristics such as gender, age, education level, occupation, and region of the populations recruited in the screening.

### 2.3 Colonoscopy and results evaluation

Colonoscopy was carried out by qualified hospitals in Tianjin. All the endoscopists involved in the screening program had extensive experience in colonoscopy. A thorough inspection was performed following the standard inspection process, and excision or biopsy was performed as needed. Endoscopic and histopathological data from colonoscopy were accurately recorded and entered into the database by the staff. Colorectal neoplasia (CN) was defined as CRC or AA or polyp; advanced colorectal neoplasia (ACN) was defined as CRC or AA; and AA was characterized as an adenoma that measured at least 1 cm in diameter, or a villous adenoma (with at least 25% villous component) or adenoma with high‐grade dysplasia. Hyperplastic polyps were not included in the definition of polyps in our study. Adenoma detection rate (ADR) was defined as the proportion of patients undergoing colonoscopy in whom at least one adenoma was detected.

### 2.4 Statistical analysis

A descriptive analysis was employed to describe the study's demographic characteristics and clinical factors. The method of univariate and multivariate logistic regression was used to analyze (1) the impact of different risk factors on the colonoscopy compliance among high‐risk individuals, and (2) the impact of different risk factors on the detection rate of CRC, AA, ACN, and CN in the high‐risk individuals.

Furthermore, in addition to our main analysis, we conducted an analysis using instrumental variables (IV). IV were initially described in econometrics and are increasingly being utilized in epidemiological and health care services research.^28^, ^29^, ^30^, ^31^, ^32^ To tackle endogeneity issues in regression analysis, we utilized probit models and the IV probit tool for comparison, enabling the estimation of the screening effect in the presence of unmeasured hidden confounders. The corresponding odds ratios (ORs) and confidence intervals (CIs) with 95% confidence were calculated for each independent risk factor. A *p*‐value <0.05 was statistically significant. All statistical analyses were conducted using R software (Version 4.1.2) and stata (Version 14).

## 3 RESULTS

### 3.1 Demographic characteristics and risk factors of high‐risk groups

In the Tianjin CRC Screening Project 2021, a total of 24,064 high‐risk individuals aged 60–74 underwent screening. However, only 5478 high‐risk individuals underwent colonoscopy examinations, resulting in an overall compliance rate of 22.76% (Figure 1). Among the 5478 individuals, 811 individuals (14.80%) tested positive on the HRFQ but did not undergo FIT testing, 957 individuals (17.47%) tested positive only on the HRFQ, 3087 individuals (56.35%) tested positive only on the FIT, and 623 individuals (11.37%) tested positive on both the HRFQ and FIT. Moreover, 4579 individuals (98.11%) underwent colonoscopy within 6 months, while 88 individuals (1.89%) had the procedure after 6 months (Table 1).

**TABLE 1:** Demographic characteristics and risk factors of high‐risk groups.

Variables		Total, *n* (%)	High‐risk population, (%)					
			High‐risk population, (%)	Colonoscopy, (%)	HRFQ(+)+No FIT	HRFQ(+)+FIT(−)	HRFQ(−)+FIT(+)	HRFQ(+)+FIT(+)
Gender	Male	29,007 9 (45.62)	10,655 (44.28)	2703 (49.34)	1375 (42.20)	4517 (40.53)	4155 (49.70)	608 (46.77)
	Female	345,767 (54.38)	13,409 (55.72)	2775 (50.66)	1883 (57.80)	6628 (59.47)	4206 (50.30)	692 (53.23)
Age	60–69	443,596 (69.76)	17,241 (71.65)	4136 (75.50)	2400 (73.66)	7891 (70.80)	5985 (71.58)	965 (74.23)
	70–74	192,255 (30.24)	6823 (28.35)	1342 (24.50)	858 (26.34)	3254 (29.20)	2376 (28.42)	335 (25.77)
Education	Elementary school above	424,526 (66.77)	18,440 (79.88)	4439 (81.03)	2517 (77.26)	8829 (79.22)	6108 (73.05)	986 (75.85)
	Elementary school/below	190,663 (29.99)	4646 (20.12)	764 (13.95)	555 (17.03)	1897 (17.02)	1978 (23.66)	216 (16.62)
Occupation	Mental work	173,786 (27.33)	5938 (24.79)	1434 (26.18)	847 (26.00)	2975 (26.69)	1798 (21.50)	318 (24.46)
	Manual work	458,897 (72.17)	18,015 (75.21)	3984 (72.73)	2358 (72.38)	8140 (73.04)	6537 (78.18)	980 (75.38)
Residential area	Central urban	201,813 (31.74)	11,589 (48.16)	3108 (56.74)	1560 (47.88)	5786 (51.92)	3457 (41.35)	786 (60.46)
	Agriculture‐related areas	431,772 (67.90)	12,475 (51.84)	2370 (43.26)	1698 (52.12)	5359 (48.08)	4904 (58.65)	514 (39.54)
History of chronic diarrhea	Yes	8089 (1.27)	3501 (14.55)	935 (17.07)	660 (20.26)	2071 (18.58)	357 (4.27)	413 (31.77)
	No	627,762 (98.73)	20,563 (85.45)	4543 (82.93)	2598 (79.74)	9074 (81.42)	8004 (95.73)	887 (68.23)
History of chronic constipation	Yes	22,082 (3.47)	5666 (23.55)	1072 (19.57)	1085 (33.30)	3488 (31.30)	623 (7.45)	470 (36.15)
	No	613,769 (96.53)	18,398 (76.45)	4406 (80.43)	2173 (66.70)	7657 (68.70)	7738 (92.55)	830 (63.85)
History of bloody mucous	Yes	2998 (0.47)	2033 (8.45)	749 (13.67)	600 (18.42)	883 (7.92)	167 (2.00)	383 (29.46)
	No	632,853 (99.53)	22,031 (91.55)	4729 (86.33)	2658 (81.58)	10,262 (92.08)	8194 (98.00)	917 (70.54)
History of chronic appendicitis or appendectomy	Yes	9262 (1.46)	2728 (11.34)	326 (5.95)	463 (14.21)	1923 (17.25)	172 (2.06)	170 (13.08)
	No	626,589 (98.54)	21,336 (88.66)	5152 (94.05)	2795 (85.79)	9222 (82.75)	8189 (97.94)	1130 (86.92)
History of chronic cholecystitis or gallstones	Yes	9890 (1.56)	3037 (12.62)	349 (6.37)	581 (17.83)	2116 (18.99)	162 (1.94)	178 (13.69)
	No	625,961 (98.44)	21,027 (87.38)	5129 (93.63)	2677 (82.17)	9029 (81.01)	8199 (98.06)	1122 (86.31)
Adverse life events	Yes	10,511 (1.65)	2386 (9.92)	237 (4.33)	434 (13.32)	1674 (15.02)	143 (1.71)	135 (10.38)
	No	625,340 (98.35)	21,678 (90.08)	5241 (95.67)	2824 (86.68)	9471 (84.98)	8218 (98.29)	1165 (89.62)
History of cancer	Yes	2793 (0.44)	2793 (11.61)	245 (4.47)	595 (18.26)	2047 (18.37)	0 (0.00)	151 (11.62)
	No	633,058 (99.56)	21,271 (88.39)	5233 (95.53)	2663 (81.74)	9098 (81.63)	8361 (100.00)	1149 (88.38)
History of polyp	Yes	3704 (0.58)	3704 (15.39)	749 (13.67)	747 (22.93)	2601 (23.34)	0 ()	356 (27.38)
	No	632,147 (99.42)	20,360 (84.61)	4729 (86.33)	2511 (77.07)	8544 (76.66)	8361 (100.00)	944 (72.62)
History of CRC in a first‐degree relative	Yes	3043 (0.48)	3043 (12.67)	496 (9.05)	640 (19.64)	2197 (19.71)	0 (0.00)	206 (15.85)
	No	632,378 (99.45)	20,983 (87.33)	4980 (90.91)	2615 (80.26)	8921 (80.04)	8355 (99.93)	1092 (84.00)
FIT	(+)	9661 (1.52)	9661 (46.43)	3710 (67.73)	—	0 (0.00)	8361 (100.00)	1300 (100.00)
	(−)	501,141 (78.81)	11,145 (53.57)	957 (17.47)	—	11,145 (100.0)	0 (0.00)	0 (0.00)
Time to colonoscopy after FIT, Mo	<6	—		4579 (83.59)	—	921 (8.26)	3042 (36.38)	616 (47.38)
	>6	—		88 (1.61)	—	36 (0.32)	45 (0.54)	7 (0.54)

*Note*: There were 5 individuals of unknown gender, 2266 individuals of residential area absence, and 125,049 individuals of no FIT among all participants; there were 20,662, 275, 186, 419, 275, and 98 individuals of education absence among all participants, colonoscopy, HRFQ(+)+No FIT, HRFQ(+)+FIT(−), HRFQ(−)+FIT(+), and HRFQ(+)+FIT(+) groups, respectively. All participants, colonoscopy, HRFQ(+)+No FIT, HRFQ(+)+FIT(−), nHRFQ(−)+FIT(+), and HRFQ(+)+FIT(+) subgroups in order of occupation absence were 3168, 60, 53, 30, 26, and 2 individuals, respectively. All participants, colonoscopy, HRFQ(+)+No FIT, HRFQ(+)+FIT(−), HRFQ(−)+FIT(+), and HRFQ(+)+FIT(+) groups had 430, 2, 3, 27, 6, and 2 individuals with a missing history of CRC in a first‐degree relative in that order; No FIT in colonoscopy 811 individuals.

Abbreviations: CRC, colorectal cancer; FIT, fecal immunochemical detection; HRFQ, high‐risk factor questionnaire; mo, months.

### 3.2 The impact of risk factors on the trends of compliance and detection rates in colonoscopy among high‐risk individuals

#### 3.2.1 Factors with high compliance and high detection rates

**FIGURE 2:**
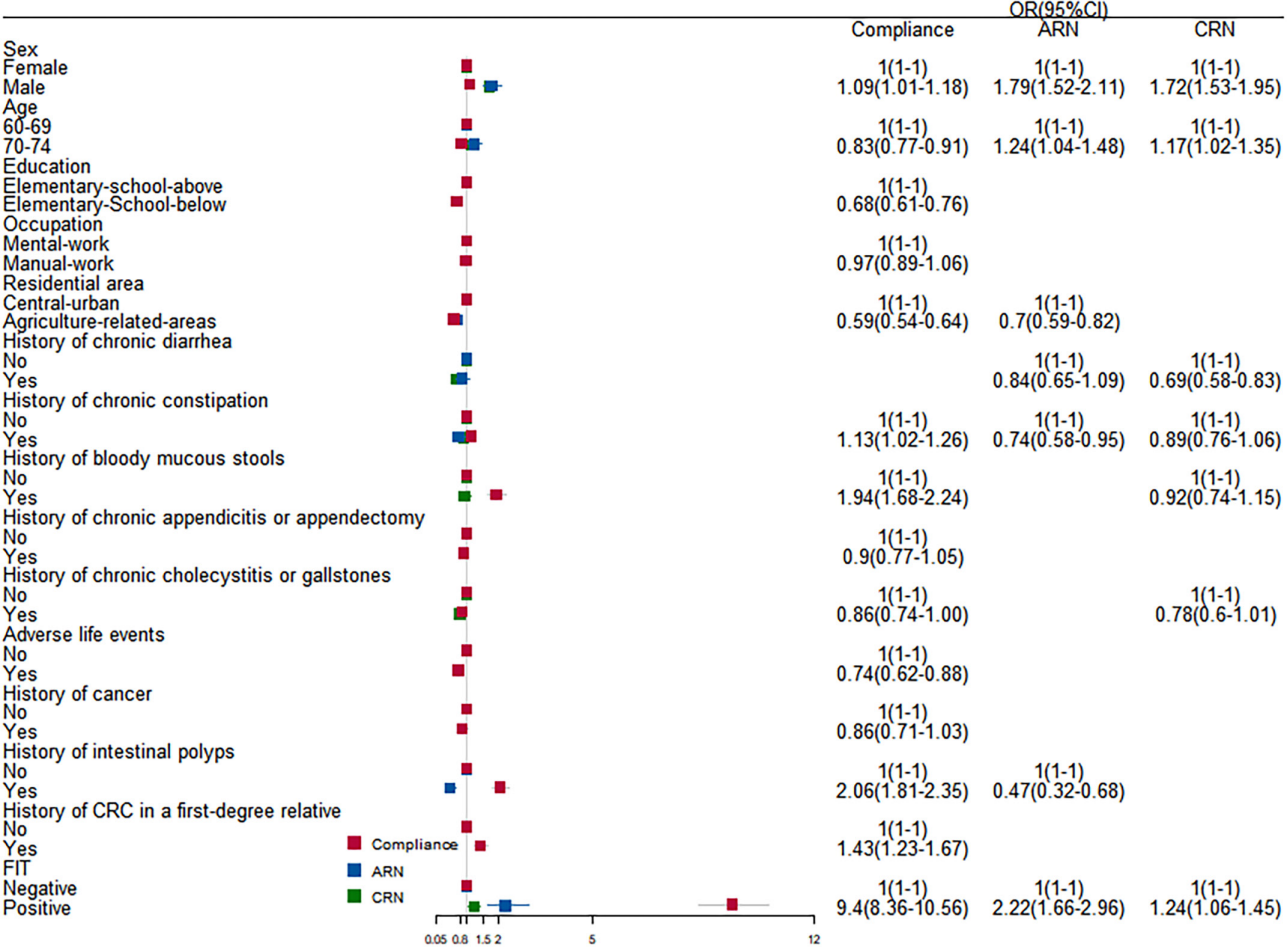
Odds ratio (OR) of risk factors associated with advanced colorectal neoplasia/colorectal neoplasm (ACN/CN) and colonoscopy compliance.

Male and individuals with a positive FIT were associated with high compliance rates and high CRC, AA, ACN, and CN detection rates (Tables S1–S5; Figure 2; Figure S1).

#### 3.2.2 Factors with low compliance and high detection rates

Individuals aged 70–74 was associated with low compliance rate but high CRC, ACN, and CN detection rates (Tables S1,S2,S4 and S5; Figure 2; Figure S1).

#### 3.2.3 Factors with low compliance and detection rates

Elementary School/below, history of chronic appendicitis or appendectomy, chronic cholecystitis or gallstones, and adverse life events were associated with low compliance rates but not associated with CRC, AA, ACN, or CN detection rates (Tables S1–S5; Figure 2; Figure S1). Agriculture‐related area was associated with low compliance rate and low CRC, AA, and ACN detection rates (Tables S1–S4; Figure 2; Figure S1). Chronic diarrhea was not associated with compliance rate but with low AA and CN detection rates (Tables S1,S3 and S5; Figure 2; Figure S1).

#### 3.2.4 Factors with high compliance and low detection rates

History of mucus blood stool, history of CRC in a first‐degree relative were associated with high compliance rates but not associated with CRC, AA, ACN, or CN detection rates (Tables S1–S5; Figure 2; Figure S1). A similar trend was found for history of chronic constipation. A history of intestinal polyps was associated with high compliance rates and low CRC, AA, and ACN detection rates, but it was not associated with the CN detection rate (Tables S1–S5; Figure 2; Figure S1).

### 3.3 Detection of intestinal tumor diseases by colonoscopy

The number of colonoscopies needed to detect one case of CRC, AA, ACN, and CN was calculated to be 29.41, 8.25, 6.44, and 1.70, respectively. CRC, AA, ACN, and CN were detected in 34, 121.2, 155.2, and 588.5 individuals of every 10,000 participants who underwent colonoscopy, respectively (Table 2).

**TABLE 2:** Detection of intestinal tumor diseases detected by colonoscopy, (n,%).

Colonoscopy	Participants taking screening colonoscopy (%)	Yield per 10,000 invitees	Colonoscopies to detect one disease (*n*)
CRC	186 (3.40)	34.0	29.41
AA	664 (12.12)	121.2	8.25
ACN	850 (15.52)	155.2	6.44
CN	3224 (58.85)	588.5	1.70

Abbreviations: AA, advanced adenomas; ACN, advanced colorectal neoplasia; CN, colorectal neoplasm; CRC, colorectal cancer.

### 3.4 Instrumental variable analyses

Using AA, CRC, and ACN as outcome variables, the results of the first stage estimation of the iv‐2sls model showed that the *p*‐values were all significant at the 1% level and the F‐statistics were all well above the empirical value of 10 (Table S8). This suggests that the instrumental variable residential area is correlated with the explanatory variable (education). Also, the *p*‐values of the AR and Wald exogeneity tests are all significant at the 1% level, indicating that the instrumental variable chosen for this paper is not a weak instrumental variable (Table S10).

The results of the IV probit models are consistent with the majority of the key results from logistic regression. There are only a few results that show some inconsistencies. Specifically, the results showed that individuals with an elementary school/below was associated with high CRC, AA, and ACN detection rates (Table S9‐S11). Additionally, history of mucus blood stool was associated with high CRC detection rate (Table S10). Seventy to 74 years was associated with a high AA detection rate (Table S9). However, chronic diarrhea was not associated with the AA detection rate (Table S9). These findings indicate that the conclusions are reliable.

## 4 DISCUSSION

Among the 5478 high‐risk individuals, the detection rates were 3.4% for CRC, 12.12% for AA, 58.85% for CN, and the ADR was 55.46%. Although the detection rate of this study is higher than other previous studies,^20^, ^23^ the compliance is still not particularly ideal, at 22.7%. It was observed that individuals aged 70–74 had a higher disease detection rate but a lower compliance. Therefore, we conducted an additional analysis to examine the influence of risk factors on compliance and detection rates in colonoscopy examinations.

It is worth noting that a positive FIT result was found to be associated with higher compliance and detection rates, which aligns with the findings of previous research studies.^6^ In addition, although some studies have suggested an association between chronic constipation and CRC,^33^, ^34^ there are also numerous inconsistent conclusions.^35^, ^36^ Our study found that history of chronic constipation is not associated with a higher detection rate of CRC.

The history of mucus blood stool and chronic diarrhea was also found to have similar findings. Clinicians paid attention to mucus blood stool as a clinical symptom of CRC.^37^, ^38^, ^39^ However, due to the ambiguity of mucus blood, relying solely on visual examination of the screening populations to determine the presence of blood in stool may be insufficient. This conclusion was further supported by subgroup analysis (Table S6). The colitis caused by diarrhea, especially non‐bloody chronic diarrhea, may be negatively associated with the occurrence of colorectal adenoma and CRC.^40^, ^41^, ^42^, ^43^ And subgroup analysis demonstrated that among individuals with a history of chronic diarrhea, the detection rate of AA and CRC was higher in those with a positive FIT compared to those with a negative FIT (Table S7). This finding aligns with the conclusions of other researchers. A similar trend was observed in the history of appendicitis surgery and gallbladder disease or gallbladder surgery. It may be due to the possible time association between the abovementioned illness histories and the increased risk of CRC.^44^, ^45^, ^46^ Considering the bias induced by lag time,^47^ the above correlation was not significant.^46^, ^48^

Considering that doctors often recommend regular colonoscopies for high‐risk individuals with colon polyps, and individuals with a history of CRC in a first‐degree relative have higher awareness of CRC, their screening compliance is better. Therefore, although a history of colon polyps and history of CRC in a first‐degree relative are important risk factors for CRC,^49^ they are not independent risk factors for CRC in this study. Equally, due to their surgical or cancer history, patients have actively or passively undergone more examinations, and some of these examinations may be routine for these diseases, such as FIT, abdominal CT, and even tumor markers. This has also provided these individuals with more opportunities to detect hidden diseases, including colorectal diseases. This proactive approach to medical intervention may contribute to a reduced incidence of intestinal polyps or CRC in this particular group, suggesting a protective factor. The presence of symptoms and personal illness history indicates that increased health awareness could enhance compliance, although its predictive value for CRC or precancerous polyps is limited.^50^, ^51^

We need to pay attention to the significant changes in people's health care‐seeking behavior, disease prevention awareness, and medical conditions as the economy and society develop. These changes have had a certain impact on the determination of relevant risk factors in the HRFQ. To the best of our knowledge, there is a limited amount of research on the impact of different high‐risk factors on compliance with colonoscopy and the detection rates of colorectal neoplastic diseases specifically within the context of the HRFQ screening strategy. Our study provides some data support for optimizing HRFQ and offers valuable evidence for decision‐making.

## 5 LIMITATIONS

Some strengths and limitations should be considered when interpreting our results. First, our data came from a large population‐based CRC screening program in China, which is a significant advantage. Furthermore, strict standards were adopted to ensure the quality of the research data. Our data were obtained from a single geographic region and cannot represent the general Chinese population. So, selection bias cannot be ruled out. Second, although the sample size was large, the compliance of colonoscopy was insufficient, and there may be deviations. Third, clinical information has not been fully obtained yet, because the follow‐up of the patients diagnosed with CRC is still in progress. Therefore, tumor stage information was not reported in our study. Finally, several factors of screening compliance were not captured: the local culture (e.g., religious beliefs), the geographical distance from home to the screening site, and others, could have an effect on participants' behavior toward CRC screening; which could be further explored in future studies.

## 6 CONCLUSIONS

This study reported several risk factors associated with the screening behaviors for CRC. Patterns and trends in CRC, AA, ACN, and CN compliance and detection rates correlate with risk factors.

## AUTHOR CONTRIBUTIONS

**Mingqing Zhang:** Conceptualization (supporting); formal analysis (equal); funding acquisition (equal); investigation (equal); methodology (equal); project administration (equal); resources (equal); validation (equal); writing – original draft (lead). **Yongdan Zhang:** Formal analysis (equal); investigation (equal); methodology (equal); writing – review and editing (equal). **Lu Guo:** Data curation (equal); software (equal). **Lizhong Zhao:** Formal analysis (equal); investigation (equal); methodology (equal); project administration (equal); writing – review and editing (supporting). **Haoren Jing:** Investigation (equal); validation (equal). **Xiao Yang:** Formal analysis (equal); validation (equal). **Wen Zhang:** Data curation (equal); software (equal). **Yong Zhang:** Data curation (equal); software (equal). **Zhenguo Nie:** Data curation (equal); investigation (equal). **Siwei Zhu:** Conceptualization (equal); visualization (equal). **Shiwu Zhang:** Conceptualization (equal); resources (equal); visualization (supporting); writing – review and editing (equal). **Xipeng Zhang:** Conceptualization (equal); funding acquisition (equal); resources (equal); supervision (equal).

## FUNDING INFORMATION

This study was funded by Foundation of Tianjin Union Medical Center (NO: 2016RMNK002, 2019ZDXK04, and 2022GCXK005). This work was funded by Foundation of Committee on Science and Technology of Tianjin (NO: 21JCYBJC01090 and 21JCZDJC00990). This work was funded by Tianjin Key Medical Discipline (Specialty) Construction Project (NO: TJYXZDXK‐044A). The funding source had no role in study design, data collection, analysis, or interpretation, report writing, or the decision to submit this paper for publication.

## CONFLICT OF INTEREST STATEMENT

The authors disclose no conflicts of interest.

## INSTITUTIONAL REVIEW BOARD STATEMENT

The study was approved by the Ethics Committee/Institutional Review Board (IRB) of Tianjin Union Medical Center (Fast Review No.C04, 2023). In addition, prior to the commencement of the study, written informed consent was obtained from all participants. All investigations and methods used were in accordance with the Declaration of Helsinki.

## Supporting information


**Data S1:**.

## Data Availability

The data, analytic methods, and study materials will not available to other researchers.
