# Supplementary material for: Trends in colorectal cancer screening compliance and incidence among 60‐ to 74‐year‐olds in China
Source: Cancer Med. 2024 Apr 17;13(8):e7133. doi: 10.1002/cam4.7133 (PMC11024507; doi:10.1002/cam4.7133)
Supplement: Supplementary file 1 — Data S1:. [file CAM4-13-e7133-s001.docx]

**Trends in colorectal cancer screening compliance and incidence Among 60-74-year-olds in China**

**Supplemental Table 1. Analysis of the influencing factors on colonoscopy compliance, (n,%)**

| Variables |  | n(%) | Unadjusted | | | | Adjusted | | | |
| --- | --- | --- | --- | --- | --- | --- | --- | --- | --- | --- |
|  |  |  | Beta | OR | 95%CI | p Value | Beta | OR | 95%CI | p Value |
| Gender | Female | 11086(55.51) | Reference |  |  |  |  |  |  |  |
|  | Male | 8886(44.49) | 0.26394 | 1.30 | 1.22-1.39 | 8.44e-15 | 0.08827 | 1.09 | 1.01-1.18 | 0.019296 |
| Age | 60-69 | 14167(70.93) | Reference |  |  |  |  |  |  |  |
|  | 70-74 | 5805(29.07) | -0.22036 | 0.80 | 0.74-0.87 | 9.98e-09 | -0.18270 | 0.83 | 0.77-0.91 | 1.60e-05 |
| Education | Elementary school above | 15893(79.58) | Reference |  |  |  |  |  |  |  |
|  | Elementary School/below | 4079(20.42) | -0.46141 | 0.63 | 0.58-0.69 | <2e-16 | -0.37967 | 0.68 | 0.61-0.76 | 2.94e-12 |
| Occupation | mental work | 4855(24.31) | Reference |  |  |  |  |  |  |  |
|  | manual work | 15117(75.69) | -0.09640 | 0.91 | 0.84-0.98 | 0.0135 | -0.03082 | 0.97 | 0.89-1.06 | 0.487196 |
| Residential area | central urban | 9483(47.48) | Reference |  |  |  |  |  |  |  |
|  | agriculture-related areas | 10489(52.52) | -0.43792 | 0.65 | 0.60-0.69 | <2e-16 | -0.52745 | 0.59 | 0.54-0.64 | < 2e-16 |
| History of chronic diarrhea | No | 17263(86.44) | Reference |  |  |  |  |  |  |  |
|  | Yes | 2709(13.56) | -0.01328 | 0.99 | 0.90-1.09 | 0.789 |  |  |  |  |
| History of chronic constipation | No | 15589(78.05) | Reference |  |  |  |  |  |  |  |
|  | Yes | 4383(21.95) | -0.49408 | 0.61 | 0.56-0.67 | <2e-16 | 0.12311 | 1.13 | 1.02-1.26 | 0.020504 |
| History of bloody mucous | No | 18627(93.27) | Reference |  |  |  |  |  |  |  |
|  | Yes | 1345(6.73) | 0.40444 | 1.50 | 1.33-1.69 | 8.53e-11 | 0.66159 | 1.94 | 1.68-2.24 | < 2e-16 |
| History of chronic appendicitis or appendectomy | No | 17789(89.07) | Reference |  |  |  |  |  |  |  |
|  | Yes | 2183(10.93) | -0.89445 | 0.41 | 0.36-0.47 | <2e-16 | -0.10230 | 0.90 | 0.77-1.05 | 0.191985 |
| History of chronic cholecystitis or gallstones | No | 17603(88.14) | Reference |  |  |  |  |  |  |  |
|  | Yes | 2369(11.86) | -0.98081 | 0.38 | 0.33-0.43 | <2e-16 | -0.15434 | 0.86 | 0.74-1.00 | 0.047433 |
| Adverse life events | No | 18081(90.53) | Reference |  |  |  |  |  |  |  |
|  | Yes | 1891(9.47) | -1.13247 | 0.32 | 0.27-0.38 | <2e-16 | -0.29990 | 0.74 | 0.62-0.88 | 0.000876 |
| History of cancer | No | 17863(89.44) | Reference |  |  |  |  |  |  |  |
|  | Yes | 2109(10.56) | -1.28116 | 0.28 | 0.24-0.33 | <2e-16 | -0.15610 | 0.86 | 0.71-1.03 | 0.094077 |
| History of Polyp | No | 17142(85.83) | Reference |  |  |  |  |  |  |  |
|  | Yes | 2830(14.17) | -0.28791 | 0.75 | 0.68-0.83 | 2.75e-08 | 0.72258 | 2.06 | 1.81-2.35 | < 2e-16 |
| History of CRC in a first-degree relative | No | 17690(88.57) | Reference |  |  |  |  |  |  |  |
|  | Yes | 2282(11.43) | -0.65724 | 0.52 | 0.46-0.59 | <2e-16 | 0.35944 | 1.43 | 1.23-1.67 | 3.30e-06 |
| FIT | (-) | 10696(53.55) | Reference |  |  |  |  |  |  |  |
|  | (+) | 9276(46.45) | 1.91869 | 6.81 | 6.29-7.38 | <2e-16 | 2.24025 | 9.40 | 8.36-10.56 | < 2e-16 |

Note: Among the 24,064 high-risk individuals, 978 individuals had missing education, 9 individuals had missing occupation, 38 individuals had missing history of bowel cancer in first-degree relatives, and 3,067 individuals had no FIT. Excluding this missing data, the data of 19,972 participants were included in the model analysis.

**Supplemental Table 2. Related factors affecting the detection of CRC in high-risk groups.**

| Variables |  | n(%) | Unadjusted | | | | Adjusted | | | |
| --- | --- | --- | --- | --- | --- | --- | --- | --- | --- | --- |
|  |  |  | Beta | OR | 95%CI | p Value | Beta | OR | 95%CI | p Value |
| Gender | Female | 60(38.96) | Reference |  |  |  |  |  |  |  |
|  | Male | 94(61.04) | 0.4823 | 1.62 | 1.17-2.25 | 0.0041 | 0.4545 | 1.58 | 1.13-2.20 | 0.00742 |
| Age | 60-69 | 99(64.29) | Reference |  |  |  |  |  |  |  |
|  | 70-74 | 55(35.71) | 0.4961 | 1.64 | 1.17-2.30 | 0.00388 | 0.4440 | 1.56 | 1.11-2.19 | 0.01036 |
| Education | Elementary school above | 139(90.26) | Reference |  |  |  |  |  |  |  |
|  | Elementary School/below | 15(9.74) | -0.51235 | 0.60 | 0.35-1.03 | 0.0625 |  |  |  |  |
| Occupation | mental work | 42(27.27) | Reference |  |  |  |  |  |  |  |
|  | manual work | 112(72.73) | -0.08354 | 0.92 | 0.64-1.32 | 0.65 |  |  |  |  |
| Residential area | central urban | 100(64.94) | Reference |  |  |  |  |  |  |  |
|  | agriculture-related areas | 54(35.06) | -0.3904 | 0.68 | 0.48-0.95 | 0.0229 | -0.4858 | 0.62 | 0.44-0.86 | 0.00498 |
| History of chronic diarrhea | No | 131(85.06) | Reference |  |  |  |  |  |  |  |
|  | Yes | 23(14.94) | 0.12717 | 1.14 | 0.72-1.78 | 0.581 |  |  |  |  |
| History of chronic constipation | No | 139(90.26) | Reference |  |  |  |  |  |  |  |
|  | Yes | 15(9.74) | -0.57876 | 0.56 | 0.33-0.96 | 0.0353 | -0.3821 | 0.68 | 0.44-0.86 | 0.17174 |
| History of bloody mucous | No | 135(87.66) | Reference |  |  |  |  |  |  |  |
|  | Yes | 19(12.34) | 0.38183 | 1.46 | 0.90-2.40 | 0.128 |  |  |  |  |
| History of chronic appendicitis or appendectomy | No | 145(94.16) | Reference |  |  |  |  |  |  |  |
|  | Yes | 9(5.84) | 0.06354 | 1.07 | 0.54-2.12 | 0.856 |  |  |  |  |
| History of chronic cholecystitis or gallstones | No | 146(94.81) | Reference |  |  |  |  |  |  |  |
|  | Yes | 8(5.19) | -0.08313 | 0.92 | 0.45-1.90 | 0.822 |  |  |  |  |
| Adverse life events | No | 149(96.75) | Reference |  |  |  |  |  |  |  |
|  | Yes | 5(3.25) | -0.18616 | 0.83 | 0.34-2.05 | 0.687 |  |  |  |  |
| History of cancer | No | 151(98.05) | Reference |  |  |  |  |  |  |  |
|  | Yes | 3(1.95) | -0.71032 | 0.49 | 0.16-1.56 | 0.227 |  |  |  |  |
| History of Polyp | No | 152(98.7) | Reference |  |  |  |  |  |  |  |
|  | Yes | 2(1.3) | -2.33637 | 0.10 | 0.02-0.39 | 0.00105 | -1.9054 | 0.15 | 0.04-0.62 | 0.00857 |
| History of CRC in a first-degree relative | No | 146(94.81) | Reference |  |  |  |  |  |  |  |
|  | Yes | 8(5.19) | -0.32921 | 0.72 | 0.35-1.48 | 0.371 |  |  |  |  |
| FIT | (-) | 8(5.19) | Reference |  |  |  |  |  |  |  |
|  | (+) | 146(94.81) | 1.5596 | 4.76 | 2.33-9.73 | 1.93e-05 | 1.1453 | 3.14 | 1.51-6.56 | 0.00227 |

Note: Among the population of 5478 routine colonoscopies, 275 participants had missing education; 2 participants had missing occupation; 2 participants had missing history of bowel cancer in first-degree relatives; and 721 participants had missing unchecked fecal occult blood. Participants with missing information were excluded from the model analysis.

**Supplemental Table 3. Related factors affecting the detection of AA in high-risk groups.**

| Variables |  | n(%) | Unadjusted | | | | Adjusted | | | |
| --- | --- | --- | --- | --- | --- | --- | --- | --- | --- | --- |
|  |  |  | Beta | OR | 95%CI | p Value | Beta | OR | 95%CI | p Value |
| Gender | Female | 222(38.14) | Reference |  |  |  |  |  |  |  |
|  | Male | 360(61.86) | 0.57384 | 1.78 | 1.48-2.12 | 3.08e-10 | 0.57578 | 1.78 | 1.48-2.13 | 4.36e-10 |
| Age | 60-69 | 416(71.48) | Reference |  |  |  |  |  |  |  |
|  | 70-74 | 166(28.52) | 0.16899 | 1.18 | 0.98-1.44 | 0.0876 |  |  |  |  |
| Education | Elementary school above | 501(86.08) | Reference |  |  |  |  |  |  |  |
|  | Elementary School/below | 81(13.92) | -0.10675 | 0.90 | 0.70-1.15 | 0.403 |  |  |  |  |
| Occupation | mental work | 159(27.32) | Reference |  |  |  |  |  |  |  |
|  | manual work | 423(72.68) | -0.09565 | 0.91 | 0.75-1.11 | 0.339 |  |  |  |  |
| Residential area | central urban | 351(60.31) | Reference |  |  |  |  |  |  |  |
|  | agriculture-related areas | 231(39.69) | -0.20608 | 0.81 | 0.68-0.97 | 0.023 | -0.29974 | 0.74 | 0.62-0.89 | 0.00114 |
| History of chronic diarrhea | No | 527(90.55) | Reference |  |  |  |  |  |  |  |
|  | Yes | 55(9.45) | -0.44790 | 0.64 | 0.48-0.86 | 0.00265 | -0.31682 | 0.73 | 0.54-0.99 | 0.04152 |
| History of chronic constipation | No | 515(88.49) | Reference |  |  |  |  |  |  |  |
|  | Yes | 67(11.51) | -0.42386 | 0.65 | 0.50-0.86 | 0.00195 | -0.23591 | 0.79 | 0.60-1.04 | 0.09579 |
| History of bloody mucous | No | 543(93.3) | Reference |  |  |  |  |  |  |  |
|  | Yes | 39(6.7) | -0.34584 | 0.71 | 0.50-1.00 | 0.0478 | -0.11364 | 0.89 | 0.62-1.28 | 0.53748 |
| History of chronic appendicitis or appendectomy | No | 556(95.53) | Reference |  |  |  |  |  |  |  |
|  | Yes | 26(4.47) | -0.2515 | 0.78 | 0.51-1.18 | 0.236 |  |  |  |  |
| History of chronic cholecystitis or gallstones | No | 557(95.7) | Reference |  |  |  |  |  |  |  |
|  | Yes | 25(4.3) | -0.31628 | 0.73 | 0.48-1.11 | 0.142 |  |  |  |  |
| Adverse life events | No | 560(96.22) | Reference |  |  |  |  |  |  |  |
|  | Yes | 22(3.78) | -0.02600 | 0.97 | 0.62-1.54 | 0.911 |  |  |  |  |
| History of cancer | No | 565(97.08) | Reference |  |  |  |  |  |  |  |
|  | Yes | 17(2.92) | -0.31319 | 0.73 | 0.44-1.22 | 0.227 |  |  |  |  |
| History of Polyp | No | 548(94.16) | Reference |  |  |  |  |  |  |  |
|  | Yes | 34(5.84) | -0.83166 | 0.44 | 0.30-0.62 | 5.68e-06 | -0.57983 | 0.56 | 0.38-0.82 | 0.00329 |
| History of CRC in a first-degree relative | No | 547(93.99) | Reference |  |  |  |  |  |  |  |
|  | Yes | 35(6.01) | -0.18691 | 0.83 | 0.58-1.19 | 0.313 |  |  |  |  |
| FIT | (-) | 58(9.97) | Reference |  |  |  |  |  |  |  |
|  | (+) | 524(90.03) | 0.9156 | 2.50 | 1.88-3.31 | 1.89e-10 | 0.65397 | 1.92 | 1.41-2.62 | 3.64e-05 |

**Supplemental Table 4.** **Related factors affecting the detection of ACN in high-risk groups.**

| Variables |  | n(%) | Unadjusted | | | | Adjusted | | | |
| --- | --- | --- | --- | --- | --- | --- | --- | --- | --- | --- |
|  |  |  | Beta | OR | 95%CI | p Value | Beta | OR | 95%CI | p Value |
| Gender | Female | 282(38.32) | Reference |  |  |  |  |  |  |  |
|  | Male | 454(61.68) | 0.58855 | 1.80 | 1.53-2.12 | 1.03e-12 | 0.58287 | 1.79 | 1.52-2.11 | 4.09e-12 |
| Age | 60-69 | 515(69.97) | Reference |  |  |  |  |  |  |  |
|  | 70-74 | 221(30.03) | 0.26476 | 1.30 | 1.09-1.55 | 0.0029 | 0.21407 | 1.24 | 1.04-1.48 | 0.0183 |
| Education | Elementary school above | 640(86.96) | Reference |  |  |  |  |  |  |  |
|  | Elementary School/below | 96(13.04) | -0.19915 | 0.82 | 0.65-1.03 | 0.0926 |  |  |  |  |
| Occupation | mental work | 201(27.31) | Reference |  |  |  |  |  |  |  |
|  | manual work | 535(72.69) | -0.09909 | 0.91 | 0.76-1.08 | 0.275 |  |  |  |  |
| Residential area | central urban | 451(61.28) | Reference |  |  |  |  |  |  |  |
|  | agriculture-related areas | 285(38.72) | -0.26273 | 0.77 | 0.65-0.90 | 0.00145 | -0.36221 | 0.70 | 0.59-0.82 | 1.75e-05 |
| History of chronic diarrhea | No | 658(89.4) | Reference |  |  |  |  |  |  |  |
|  | Yes | 78(10.6) | -0.31747 | 0.73 | 0.57-0.94 | 0.0136 | -0.17617 | 0.84 | 0.65-1.09 | 0.1848 |
| History of chronic constipation | No | 654(88.86) | Reference |  |  |  |  |  |  |  |
|  | Yes | 82(11.14) | -0.48099 | 0.62 | 0.48-0.79 | 0.000119 | -0.29886 | 0.74 | 0.58-0.95 | 0.0205 |
| History of bloody mucous | No | 678(92.12) | Reference |  |  |  |  |  |  |  |
|  | Yes | 58(7.88) | -0.15553 | 0.86 | 0.64-1.14 | 0.294 |  |  |  |  |
| History of chronic appendicitis or appendectomy | No | 701(95.24) | Reference |  |  |  |  |  |  |  |
|  | Yes | 35(4.76) | -0.18469 | 0.83 | 0.58-1.20 | 0.324 |  |  |  |  |
| History of chronic cholecystitis or gallstones | No | 703(95.52) | Reference |  |  |  |  |  |  |  |
|  | Yes | 33(4.48) | -0.27599 | 0.76 | 0.52-1.10 | 0.149 |  |  |  |  |
| Adverse life events | No | 709(96.33) | Reference |  |  |  |  |  |  |  |
|  | Yes | 27(3.67) | -0.06405 | 0.94 | 0.62-1.43 | 0.764 |  |  |  |  |
| History of cancer | No | 716(97.28) | Reference |  |  |  |  |  |  |  |
|  | Yes | 20(2.72) | -0.40904 | 0.66 | 0.41-1.07 | 0.0902 |  |  |  |  |
| History of Polyp | No | 700(95.11) | Reference |  |  |  |  |  |  |  |
|  | Yes | 36(4.89) | -1.06078 | 0.35 | 0.24-0.49 | 2.38e-09 | -0.76172 | 0.47 | 0.32-0.68 | 5.85e-05 |
| History of CRC in a first-degree relative | No | 693(94.16) | Reference |  |  |  |  |  |  |  |
|  | Yes | 43(5.84) | -0.22975 | 0.79 | 0.57-1.11 | 0.175 |  |  |  |  |
| FIT | (-) | 66(8.97) | Reference |  |  |  |  |  |  |  |
|  | (+) | 670(91.03) | 1.0717 | 2.92 | 2.24-3.80 | 1.9e-15 | 0.79734 | 2.22 | 1.66-2.96 | 5.49e-08 |

**Supplemental Table 5. Related factors affecting the detection of CN in high-risk groups.**

| Variables |  | n(%) | Unadjusted | | | | Adjusted | | | |
| --- | --- | --- | --- | --- | --- | --- | --- | --- | --- | --- |
|  |  |  | Beta | OR | 95%CI | p Value | Beta | OR | 95%CI | p Value |
| Gender | Female | 1209(44.88) | Reference |  |  |  |  |  |  |  |
|  | Male | 1485(55.12) | 0.56134 | 1.75 | 1.55-1.98 | < 2e-16 | 0.54435 | 1.72 | 1.53-1.95 | < 2e-16 |
| Age | 60-69 | 1966(72.98) | Reference |  |  |  |  |  |  |  |
|  | 70-74 | 728(27.02) | 0.18447 | 1.20 | 1.05-1.38 | 0.00906 | 0.15719 | 1.17 | 1.02-1.35 | 0.02859 |
| Education | Elementary school above | 2290(85) | Reference |  |  |  |  |  |  |  |
|  | Elementary School/below | 404(15) | -0.01516 | 0.98 | 0.83-1.16 | 0.859 |  |  |  |  |
| Occupation | mental work | 704(26.13) | Reference |  |  |  |  |  |  |  |
|  | manual work | 1990(73.87) | -0.05651 | 0.95 | 0.82-1.08 | 0.42 |  |  |  |  |
| Residential area | central urban | 1477(54.83) | Reference |  |  |  |  |  |  |  |
|  | agriculture-related areas | 1217(45.17) | 0.11370 | 1.12 | 0.99-1.26 | 0.0649 |  |  |  |  |
| History of chronic diarrhea | No | 2387(88.6) | Reference |  |  |  |  |  |  |  |
|  | Yes | 307(11.4) | -0.43206 | 0.65 | 0.55-0.77 | 9.03e-07 | -0.36976 | 0.69 | 0.58-0.83 | 6.39e-05 |
| History of chronic constipation | No | 2301(85.41) | Reference |  |  |  |  |  |  |  |
|  | Yes | 393(14.59) | -0.24668 | 0.78 | 0.66-0.92 | 0.00275 | -0.11145 | 0.89 | 0.76-1.06 | 0.19592 |
| History of bloody mucous | No | 2477(91.95) | Reference |  |  |  |  |  |  |  |
|  | Yes | 217(8.05) | -0.25377 | 0.78 | 0.63-0.95 | 0.0163 | -0.08447 | 0.92 | 0.74-1.15 | 0.45295 |
| History of chronic appendicitis or appendectomy | No | 2553(94.77) | Reference |  |  |  |  |  |  |  |
|  | Yes | 141(5.23) | -0.13435 | 0.87 | 0.67-1.13 | 0.31 |  |  |  |  |
| History of chronic cholecystitis or gallstones | No | 2566(95.25) | Reference |  |  |  |  |  |  |  |
|  | Yes | 128(4.75) | -0.39508 | 0.67 | 0.52-0.87 | 0.0024 | -0.24782 | 0.78 | 0.60-1.01 | 0.06413 |
| Adverse life events | No | 2597(96.4) | Reference |  |  |  |  |  |  |  |
|  | Yes | 97(3.6) | -0.17506 | 0.84 | 0.62-1.14 | 0.263 |  |  |  |  |
| History of cancer | No | 2596(96.36) | Reference |  |  |  |  |  |  |  |
|  | Yes | 98(3.64) | -0.12239 | 0.88 | 0.65-1.21 | 0.438 |  |  |  |  |
| History of Polyp | No | 2377(88.23) | Reference |  |  |  |  |  |  |  |
|  | Yes | 317(11.77) | 0.03791 | 1.04 | 0.86-1.25 | 0.692 |  |  |  |  |
| History of CRC in a first-degree relative | No | 2505(92.98) | Reference |  |  |  |  |  |  |  |
|  | Yes | 189(7.02) | 0.00136 | 1.00 | 0.79-1.27 | 0.991 |  |  |  |  |
| FIT | (-) | 484(17.97) | Reference |  |  |  |  |  |  |  |
|  | (+) | 2210(82.03) | 0.33450 | 1.40 | 1.21-1.62 | 8.47e-06 | 0.21404 | 1.24 | 1.06-1.45 | 0.00712 |

**Supplemental Table 6**. **Detection of intestinal tumor diseases in positive/negative FIT of people with history of bloody mucous.**

|  |  | n | Colonoscopy | CN | ACN | CRC | AA | Polyp | Normal |
| --- | --- | --- | --- | --- | --- | --- | --- | --- | --- |
| History of bloody mucous (+) | FIT (+) | 550 | 241 | 143(59.34) | 45(18.67) | 16（6.64） | 29（12.03） | 98（40.66） | 98 (40.66) |
|  | FIT (-) | 1615 | 185 | 91(49.19) | 19(10.27) | 5（2.70） | 14（7.57） | 72（38.92） | 94（50.81） |
| History of bloody mucous (-) | FIT (+) | 9111 | 3469 | 2151(62.01) | 652(18.80) | 134（3.86） | 518（14.93） | 149（42.88） | 1318 (37.99) |
|  | FIT (-) | 499526 | 2214 | 1271(57.41) | 289(13.05) | 99（4.47） | 190（8.58） | 982(44.35） | 943（42.59） |

**Supplemental Table 7. Detection of intestinal tumor diseases in positive/negative FIT of people with history of chronic diarrhoea.**

|  |  | n | Colonoscopy | CN | ACN | CRC | AA | Polyp | Normal |
| --- | --- | --- | --- | --- | --- | --- | --- | --- | --- |
| History of chronic diarrhea (+) | FIT (+) | 770 | 386 | 211(54.66) | 64(16.58) | 19（4.92） | 45（11.66） | 147（38.08） | 175（45.34） |
|  | FIT (-) | 5929 | 266 | 124(46.62) | 19(7.14) | 6（2.26） | 13（4.89） | 105（39.47） | 142（53.38） |

Using AA, CRC and ACN as outcome variables, the results of the first stage estimation of the iv-2sls model showed that the P-values were all significant at the 1% level and the F-statistics were all well above the empirical value of 10. This suggests that the instrumental variable residential area is correlated with the explanatory variable (education). Also, the *P*-values of the AR and Wald exogeneity tests are all significant at the 1% level, indicating that the instrumental variable chosen for this paper is not a weak instrumental variable(Table S10).

**Supplemental Table 8. First stage model estimation results**

| Variables | ACN | CN | CRC | AA |
| --- | --- | --- | --- | --- |
| First stage F-statistic | 58.14 | 58.14 | 58.14 | 58.14 |
| P | 0.0000 | 0.0000 | 0.0000 | 0.0000 |
| Wald | 16.33 | 2.20 | 6.80 | 8.97 |
| P | 0.0001 | 0.1377 | 0.0091 | 0.0027 |
| AR | 16.66 | 2.21 | 6.83 | 9.09 |
| P | 0.0000 | 0.1369 | 0.0090 | 0.0026 |
| Sample size | 4478 | 4478 | 4478 | 4478 |

**Supplementary Table 9. Probit models and phase II IV probit models, variable-adjusted* estimates of AA**

| Variables |  | **Probit** | | | **IV-Probit** | | |
| --- | --- | --- | --- | --- | --- | --- | --- |
|  |  | B | 95%CI | p Value | Beta | 95%CI | p Value |
| Gender | Female |  |  |  |  |  |  |
|  | Male | 0.29926 | 0.20,0.40 | 0.000 | 0.27070 | 0.17,0.37 | 0.000 |
| Age | 60-69 |  |  |  |  |  |  |
|  | 70-74 | 0.07939 | -0.03,0.19 | 0. 152 | 0.11985 | 0.01,0.23 | 0.035 |
| Education | Elementary school above |  |  |  |  |  |  |
|  | Elementary School/below | 0. 07283 | -0.07,0.21 | 0.305 | 0.55537 | 0.20,0.91 | 0.002 |
| Occupation | mental work |  |  |  |  |  |  |
|  | manual work | 0. 04663 | -0.06,0.16 | 0. 405 | -0.01885 | -0.14,0.10 | 0.755 |
| History of chronic diarrhea | No |  |  |  |  |  |  |
|  | Yes | -0. 16880 | -0.33, -0.01 | 0.036 | -0.15579 | -0.31,0.00 | 0.051 |
| History of chronic constipation | No |  |  |  |  |  |  |
|  | Yes | -0.12621 | -0.27,0.02 | 0.088 | -0.12883 | -0.27,0.01 | 0.078 |
| History of bloody mucous | No |  |  |  |  |  |  |
|  | Yes | -0.04537 | -0.24,0.15 | 0. 641 | -0.04740 | -0.24,0.14 | 0.621 |
| History of chronic appendicitis or appendectomy | No |  |  |  |  |  |  |
|  | Yes | -0.01319 | -0.24,0.21 | 0.909 | -0.01808 | -0.24,0.21 | 0.874 |
| History of chronic cholecystitis or gallstones | No |  |  |  |  |  |  |
|  | Yes | -0.02447 | -0.25,0.20 | 0.832 | -0.03728 | -0.26,0.19 | 0.746 |
| Adverse life events | No |  |  |  |  |  |  |
|  | Yes | 0.12047 | -0.13,0.37 | 0.352 | 0.12955 | -0.12,0.38 | 0.316 |
| History of cancer | No |  |  |  |  |  |  |
|  | Yes | 0.04615 | -0.23,0.32 | 0.742 | 0.01465 | -0.26,0.29 | 0.917 |
| History of Polyp | No |  |  |  |  |  |  |
|  | Yes | -0.27196 | -0.46, -0.09 | 0.004 | -0.28613 | -0.47,-0.10 | 0.002 |
| History of CRC in a first-degree relative | No |  |  |  |  |  |  |
|  | Yes | 0.08196 | -0.13,0.29 | 0. 445 | 0.04516 | -0.17,0.26 | 0.676 |
| FIT | (-) |  |  |  |  |  |  |
|  | (+) | 0.36323 | 0.19,0.53 | 0.000 | 0.37924 | 0.21,0.55 | 0.000 |

Note: Probit Model Wald chi2=110.67, Model Wald *P*=0.0000; IV-Probit Model Wald chi2= 7.68, Model Wald *P*= 0.0056.

**Supplementary Table 10. Probit models and phase II IV probit models, variable-adjusted* estimates of CRC**

| Variables |  | Probit | | | IV-Probit | | |
| --- | --- | --- | --- | --- | --- | --- | --- |
|  |  | B | 95%CI | p Value | Beta | 95%CI | p Value |
| Gender | Female |  |  |  |  |  |  |
|  | Male | 0. 17710 | 0.03,0.32 | 0.016 | 0. 74611 | 0.20,1.29 | 0.007 |
| Age | 60-69 |  |  |  |  |  |  |
|  | 70-74 | 0. 23649 | 0.08,0.39 | 0.003 | 0. 15273 | 0.01,0.30 | 0. 037 |
| Education | Elementary school above |  |  |  |  |  |  |
|  | Elementary School/below | 0. 29302 | 0.06,0.52 | 0.012 | 0. 27101 | 0.11,0.43 | 0. 001 |
| Occupation | mental work |  |  |  |  |  |  |
|  | manual work | -0. 01687 | -0.18,0.15 | 0. 841 | -0. 07583 | -0.26,0.10 | 0. 408 |
| History of chronic diarrhea | No |  |  |  |  |  |  |
|  | Yes | 0. 11152 | -0.11,-0.36 | 0. 330 | 0. 11862 | -0.10,0.34 | 0. 292 |
| History of chronic constipation | No |  |  |  |  |  |  |
|  | Yes | -0. 18676 | -0.43,0.05 | 0.126 | -0. 18481 | -0.42,0.05 | 0. 126 |
| History of bloody mucous | No |  |  |  |  |  |  |
|  | Yes | 0. 30101 | 0.05,0.55 | 0.019 | 0. 29009 | 0.04,0.54 | 0. 022 |
| History of chronic appendicitis or appendectomy | No |  |  |  |  |  |  |
|  | Yes | 0. 11998 | -0.19,0.43 | 0. 452 | 0. 11337 | -0.19,0.42 | 0. 471 |
| History of chronic cholecystitis or gallstones | No |  |  |  |  |  |  |
|  | Yes | 0. 05938 | -0.27,0.39 | 0. 724 | 0. 04917 | -0.28,0.38 | 0. 769 |
| Adverse life events | No |  |  |  |  |  |  |
|  | Yes | -0. 06416 | -0.47,0.34 | 0. 755 | -0. 05493 | -0.45,0.34 | 0. 787 |
| History of cancer | No |  |  |  |  |  |  |
|  | Yes | -0. 02240 | -0.50,0.46 | 0. 927 | -0. 06009 | -0.53,0.41 | 0. 804 |
| History of Polyp | No |  |  |  |  |  |  |
|  | Yes | -0. 73874 | -1.22,-0.25 | 0.003 | -0. 73410 | -1.21,0.26 | 0. 003 |
| History of CRC in a first-degree relative | No |  |  |  |  |  |  |
|  | Yes | 0. 06449 | -0.27,0.40 | 0. 709 | 0. 02695 | -0.31,0.36 | 0. 874 |
| FIT | (-) |  |  |  |  |  |  |
|  | (+) | 0. 58546 | 0.29,0.89 | 0.000 | 0. 60135 | 0.31,0.90 | 0.000 |

Note: Probit Model Wald chi2=58.32, Model Wald *P*=0.0000; IV-Probit Model Wald chi2=3.25, Model Wald *P*=0.0713.

**Supplementary Table 11. Probit models and phase II IV probit models, variable-adjusted* estimates of ACN**

| Variables |  | Probit | | | IV-Probit | | |
| --- | --- | --- | --- | --- | --- | --- | --- |
|  |  | B | 95%CI | p Value | Beta | 95%CI | p Value |
| Gender | Female |  |  |  |  |  |  |
|  | Male | 0. 31305 | 0.22, 0.40 | 0.000 | 0.27861 | 0.19,0.37 | 0.000 |
| Age | 60-69 |  |  |  |  |  |  |
|  | 70-74 | 0.14259 | 0.04, 0.24 | 0.006 | 0.18820 | 0.08,0.29 | 0.000 |
| Education | Elementary school above |  |  |  |  |  |  |
|  | Elementary School/below | 0.14287 | 0.01, 0.28 | 0.035 | 0.70718 | 0.38,1.03 | 0.000 |
| Occupation | mental work |  |  |  |  |  |  |
|  | manual work | 0. 03625 | -0.07, 0.14 | 0.493 | -0.04001 | -0.15,0.07 | 0.481 |
| History of chronic diarrhea | No |  |  |  |  |  |  |
|  | Yes | -0. 10627 | -0.25, 0.04 | 0.153 | -0.09230 | -0.24,0.05 | 0.209 |
| History of chronic constipation | No |  |  |  |  |  |  |
|  | Yes | -0. 16336 | -0.30,-0.03 | 0.020 | -0.16438 | -0.30,-0.03 | 0.018 |
| History of bloody mucous | No |  |  |  |  |  |  |
|  | Yes | 0. 06327 | -0.11, 0.24 | 0. 474 | 0.05704 | -0.11,0.23 | 0.513 |
| History of chronic appendicitis or appendectomy | No |  |  |  |  |  |  |
|  | Yes | 0. 02806 | -0.18, 0.24 | 0. 792 | 0.02042 | -0.19,0.23 | 0.846 |
| History of chronic cholecystitis or gallstones | No |  |  |  |  |  |  |
|  | Yes | -0. 00578 | -0.22, 0.20 | 0.957 | -0.02067 | -0.23,0.19 | 0.846 |
| Adverse life events | No |  |  |  |  |  |  |
|  | Yes | 0. 08666 | -0.15, 0.33 | 0.482 | 0.09724 | -0.14,0.34 | 0.430 |
| History of cancer | No |  |  |  |  |  |  |
|  | Yes | 0. 03321 | -0.23, 0.30 | 0.805 | -0.00510 | -0.27,0.26 | 0.970 |
| History of Polyp | No |  |  |  |  |  |  |
|  | Yes | -0. 37179 | -0.55, -0.19 | 0.000 | -0.38433 | -0.56,-0.20 | 0.000 |
| History of CRC in a first-degree relative | No |  |  |  |  |  |  |
|  | Yes | 0. 08736 | -0.11, 0.29 | 0.390 | 0.04348 | -0.16,0.24 | 0.669 |
| FIT | (-) |  |  |  |  |  |  |
|  | (+) | 0. 45616 | 0.30, 0.62 | 0.000 | 0.47286 | 0.31,0.63 | 0.000 |

Note: Probit Model Wald chi2=154.61，Model Wald P=0.0000; IV-Probit Model Wald chi2=12.19，Model Wald P=0.0005.

**
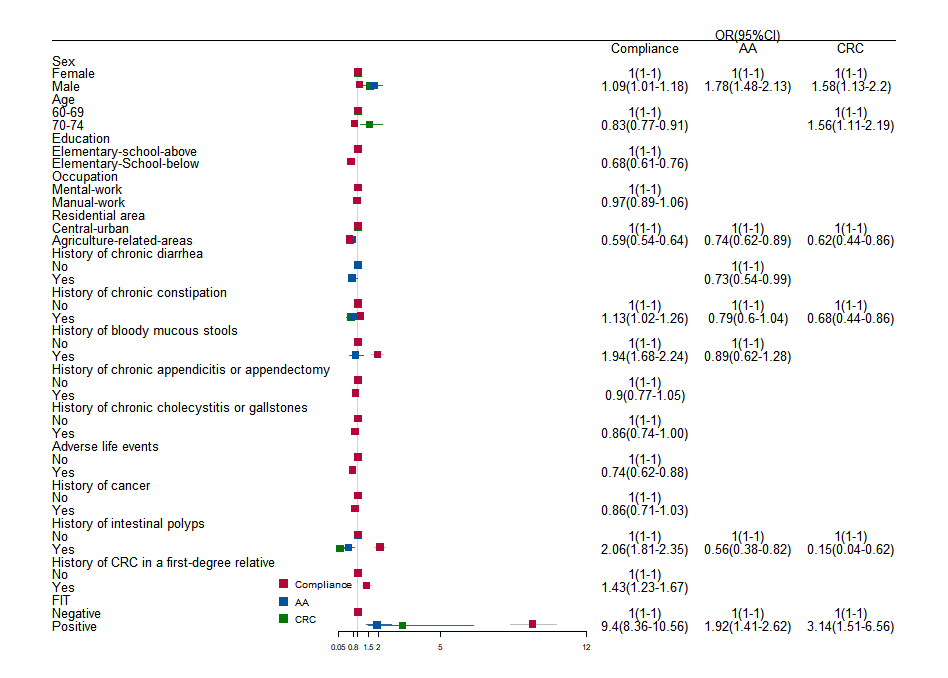
**

**Supplemental Figure 1.** OR of risk factors associated with CRC/AAand colonoscopy compliance.
